# Biochemical, structural characterization and *in-vitro* evaluation of antioxidant, antibacterial, cytotoxic, and antidiabetic activities of nanosuspensions of *Cinnamomum zeylanicum* bark extract

**DOI:** 10.3389/fchem.2023.1194389

**Published:** 2023-05-05

**Authors:** Aqsa Nawaz, Tayyab Ali, Muhammad Naeem, Fatma Hussain, Zhiye Li, Abdul Nasir

**Affiliations:** ^1^ Clinico-Molecular Biochemistry Laboratory, Department of Biochemistry, Faculty of Sciences, University of Agriculture, Faisalabad, Pakistan; ^2^ College of Life Science, Hebei Normal University, Shijiazhuang, China; ^3^ Department of Pharmacy, Second Affiliated Hospital of Zhengzhou University, Zhengzhou, Henan, China; ^4^ Medical Research Center, Second Affiliated Hospital of Zhengzhou University, Zhengzhou, Henan, China

**Keywords:** nanosuspensions, alpha-amylase inhibition, therapeutic efficacy, antidiabetic potential, antioxidant potential

## Abstract

*Cinnamomum zeylanicum* is a traditional medicinal plant known for its anti-inflammatory, antidiabetic, antimicrobial, anticancer, and antioxidant properties. Its therapeutic efficacy using nanosuspensions is still unclear for treating infectious diseases. This study was designed to evaluate the bioactivities, biochemical characterization, and bioavailability of freshly prepared nanosuspensions of *C. zeylanicum*. Structural and biochemical characterization of *C. zeylanicum* and its biological activities, such as antioxidants, antimicrobials, antiglycation, α-amylase inhibition, and cytotoxicity was performed using Fourier-transform infrared (FTIR) spectroscopy and High-Performance Liquid Chromatography (HPLC). *C. zeylanicum* extract and nanosuspensions showed TPCs values of 341.88 and 39.51 mg GAE/100 g while showing TFCs as 429.19 and 239.26 mg CE/100g, respectively. DPPH inhibition potential of *C. zeylanicum* extract and nanosuspension was 27.3% and 10.6%, respectively. Biofilm inhibition activity revealed that bark extract and nanosuspension showed excessive growth restraint against *Escherichia coli*, reaching 67.11% and 66.09%, respectively. The α-amylase inhibition assay of extract and nanosuspension was 39.3% and 6.3%, while the antiglycation activity of nanosuspension and extract was 42.14% and 53.76%, respectively. Extracts and nanosuspensions showed maximum hemolysis at 54.78% and 19.89%, respectively. Results indicated that nanosuspensions possessed antidiabetic, antimicrobial, anticancer, and antioxidant properties. Further study, however, is needed to assess the clinical studies for the therapeutic use of nanosuspensions.

## Graphical Abstract

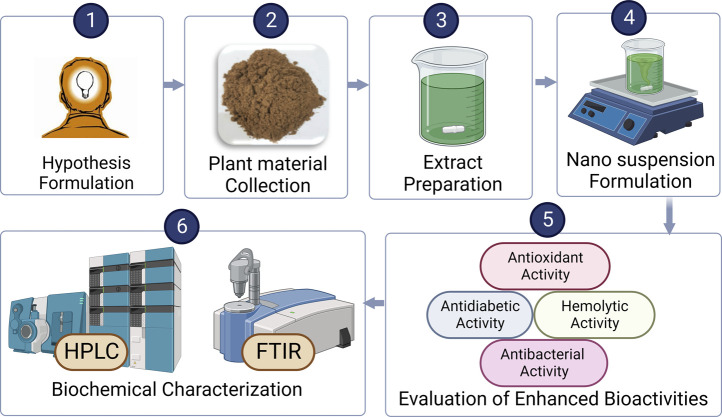

## 1 Introduction

Different pharmaceutical drugs and chemical compounds have been widely and extensively used to treat infectious and inflammatory disorders (Rahman et al., 2022). Excessive use of these medicines negatively affects vital organs and tissues, resulting in cellular toxicity (Johnson et al., 2022). For example, digoxin increases the risk of digestive and cardiovascular problems; acetaminophen increases the development of hepatic carcinoma, and carbamazepine enhances the nystagmus risk and blood dyscrasias (Sahar et al., 2022). Medicinal plants are used as alternative phytomedicines to synthetic medicines to treat various disorders, cancers, and inflammatory diseases (Gahtori et al., 2023). These medicinal plants are a rich source of bioactive compounds, including tannins, alkaloids, steroids, flavonoids, resins, fatty acids and other derived substances. These bioactive components in plant extracts make them more valuable for therapeutic applications (Jain et al., 2019).

Cinnamon (*Cinnamomum zeylanicum*) belongs to the Lauraceae family, widely used as herbal medicine due to its wide range of therapeutic effects (Hussain et al., 2019). Cinnamon is a rich source of calcium, manganese, iron and dietary fibers. It contains a diverse range of bioactive like cinnamaldehyde, cinnamic acid, cinnamate, polyphenols and antioxidants responsible for their anti-inflammatory, antidiabetic, antibacterial, and anticancer activities (Sharifi-Rad et al., 2021). Cinnamon essential oils and phenolic components are beneficial for human health. Cinnamon effectively cures diabetes, arteriosclerosis, arthritis, and Alzheimer’s disease (Kowalska et al., 2021).

Different studies in the literature revealed the differential formulation of nanosuspensions in various plants such as *Piper nigrum* (Zafar et al., 2019), *Coriandrum sativum* (Jahan et al., 2016), *Terminalia arjuna* (Zafar et al., 2020). However, no studies have been reported yet in the literature on the synthesis of *C. zeylanicum* nanosuspensions and enhanced bioactivities. As a result, there is a need to investigate the biochemical characterization of different bioactive components responsible for the improved bioactivities and bioavailability of *C. zeylanicum* nanosuspensions.

Recent advances in nanotechnology have led to the development of nanomedicines and nanosuspensions, which offer several advantages such as improved drug delivery, reduced toxicity, and increased bioavailability (Ma et al., 2023). These nano-based medicines are cost-effective and possess high efficacy against a variety of different diseases (Ali et al., 2022). Nowadays, nanosuspensions have gained a special interest in drug delivery due to their small size, high stability, water solubility and higher bioavailability than synthetic medicines (Grifoni et al., 2022). Nanosuspension preparation is highly cost-effective and reliable than the traditional methods for drug design and can be used to deliver of water-insoluble drugs. Different methods and techniques are currently applied for synthesizing nanosuspensions, including wet milling, high-pressure emulsion, solvent evaporation, and emulsification (Jacob et al., 2020).

Nanosuspensions possess several advantages over traditional pharmaceutical ingredients due to improved bioavailability for oral drug administration, high dissolution rate and enhanced penetration rate to the skin surface (Ma et al., 2023). Nanosuspensions are also employed in the pharmaceutical industry for drug delivery that were evenly distributed over the skin surface leading to a high concentration gradient. It was reported that patients with skin diseases required long-term medications that caused serious side effects. Therefore, nanosuspensions technology has improved the success rate in patients with skin diseases through enhanced skin surface penetration that required long-term medications and increase therapeutic effect (Oktay et al., 2018).

We hypothesized that the nanosuspensions could lead to enhanced bioactivities than extract due to the enhanced bioavailability of phytoconstituents. For improved bioactivities, we created nanosuspensions from *C. zeylanicum* bark extract. Both the extract and nanosuspensions were assessed biochemically. These nanosuspensions formation may open the way for further research into the improved availability of plant-based medicinal substances.

## 2 Materials and methods

### 2.1 Chemicals and reagents

Different analytical grade chemicals and reagents were used in this study. These included the acetonitrile, ethanol, acetone, alpha-amylase, DDPH (2,2, Diphenyl-1- picrylhydrazyl) and Folin Ciocalteu reagent (Sigma Aldrich Taufkirchen Germany), PVA (polyvinyl alcohol) (Appli.Chem, United States), BSA (Bovine serum albumin) (Merck Darmstadt, Germany). All standards (quercetin, chlorogenic acids, p-coumaric, gallic acid and vanillic acid) were provided by Sigma Aldrich. Chemicals and solvents for the HPLC analysis were purchased from Merck.

### 2.2 Collection and preparation of plant extracts

Cinnamon barks were collected from the market and examined by the botanist. After drying, *C. zeylanicum* was grounded into powder and kept in a clean, air-tight vessel or jar at room temperature. Extraction was carried out by Soxhlet apparatus using 95% ethanol as a solvent and the extract was separated by filtration. Then, the extract was placed in the refrigerator for use in further subsequential experiments (Mishra et al., 2013).

### 2.3 Synthesis of nanosuspensions

Nanosuspensions of *C. zeylanicum* were prepared by following the nanoprecipitation technique. Following standard protocols, 1 g of cinnamon extract was dissolved into 6 mL of acetone and ethanol (3:1) solution and mixed into 10 mL of water, containing 1.5% w/v polyvinyl alcohol (PVA) with repetitively magnetically stirred at 1,000 rpm for 30 min. The resultant mixture was diluted in 20 mL PVA to reduce the coalescence. The solution was stirred at 500 rpm for 6 h at 25°C for solvent evaporation. Finally, nanosuspensions were formed and frozen at −18°C in a refrigerator (Ali et al., 2022).

### 2.4 Antioxidant activity

The antioxidant profile of *C. zeylanicum* was assessed by the following methods.

#### 2.4.1 Total phenolic content (TPCs)

Total phenolic contents were accessed through the Folin-Ciocalteu reagent method. In this method, the reaction mixture was prepared by dissolving the 100 µL Na_2_CO_3_ solution, test samples (125 µL) and diluted reagent (10 percent; 25 µL) kept for incubation at 25°C for 60 min. After that, absorbance was measured by the spectrophotometer at 765 nm. The presence of blue color showed the existence of phenolic components in nanosuspensions (Chahardehi et al., 2009).

#### 2.4.2 Total flavonoid content (TFCs)

Total flavonoid contents were accessed through the AlCl_3_ colorimetric method. The solution was prepared by dissolving the 9.5 µL of NaNO_2,_ 38 µL of test samples and 156 µL of distilled water in 96 well plates and was incubated at room temperature for 10 min. Then, 19 µL of 10% of AlCl_3_ was mixed with the reaction mixture and incubated at room temperature for 5 min. Finally, absorbance was measured by a spectrophotometer at 510 nm (Sahar et al., 2022).

#### 2.4.3 DPPH free radical scavenging assay

The antioxidant activity of nanosuspensions was accessed by DPPH radical scavenging assay. Following this method, 250 µL of DPPH solution (0.004 mg DPPH in 100 mL methanol) was mixed with 2.5 µL of extract and nanosuspensions and covered with aluminum foil. Absorbance was recorded by spectrophotometer at 520 nm (Hussain et al., 2021). The radical scavenging assay was measured by using the given formula:

$$\%\;DPPH\;scavenging=\left[A\;\left(control\right)\;–\;A\;\left(sample\right)\;/A\;\left(control\right)\right]\times 100$$

### 2.5 Biofilm formation inhibition assay

Antibacterial activity was evaluated through a biofilm inhibition assay. The solution mixture was prepared by mixing the nutrient broth, sample, and 100 µL of *Escherichia coli* and *Staphylococcus aureus* in the 96-well tissue culture microliter plate for incubation aerobically at 37°C overnight. Plates were washed with PBS (pH = 7.4) three times and kept for air drying. Then, 100 µL of crystal violet stain (50%) was applied to the reaction mixture and the excess stain was washed away with tap water. Then, the dye was then mixed with 100 µL glacial acetic acid (33% v/v). The microplate reader (BioTek, United States) was used to measure the absorbance at 630 nm (Hussain et al., 2021). Ciprofloxacin was used as a positive control, while the negative control was nutrient broth along bacterial strains. Percentage inhibition by using the given formula:

$$\%\;Biofilm\;inhibition=\left[A\;\left(control\right)\;–\;A\;\left(sample\right)\;/A\;\left(control\right)\right]\times 100$$

### 2.6 Cytotoxic activity

The hemolytic activity of extract and nanosuspensions was accessed through an ELISA microtiter plate. Briefly, 3 mL of blood was centrifuged at 8,000 rpm for almost 5 min. The supernatant plasma was disposed of, and pellets of red blood cells were washed three times with 5 mL PBS saline and centrifugated for 5 min at 8,000 rpm. Then, 200 µL of chilled tubular contents was added to the prepared mixture and hemolytic activity was determined using an ELISA microtiter plate at 570 nm. Triton X-100 was utilized as a positive control and PBS was used negatively as a negative control (BioTek, Winooski, VT, United States) (Powell et al., 2000).

$$\%\;Hemolysis=A\;\left(sample\right)-A\;\left(negative\;control\right)/A\;\left(positive\;control\right)-A\;\left(negative\;control\right)\times 100$$

### 2.7 Antidiabetic evaluation

#### 2.7.1 Antiglycation potential

The solution for the antiglycation assay was prepared by dissolving D-glucose and BSA in sodium phosphate buffer and stored at 37°C for 2 days. Absorbance was determined using the spectrophotometer at different wavelengths (BMS UV-2600, Japan). The solution lacking D-glucose was used as a control. Synthetic metformin was used as a reference component (Matsuda et al., 2003).

$$\%\;Antiglycation\;potential=\left[A\;\left(440\;nm\right)\;/\;A\;\left(370\;nm\right)-A\;\left(440\;nm\right)\right]\times 100$$

#### 2.7.2 Alpha-amylase inhibition assay

The antiglycation potential of nanosuspensions and extract was determined through an alpha-amylase inhibition assay. Samples were kept in a 96-well plate at room temperature for 10 min before being treated with an amylase solution in sodium phosphate. In the end, the solution of iodine was mixed with the reaction mixture. Absorbance was determined using the spectrophotometer compared to a blank solution at 630 nm (Oshiomame Unuofin et al., 2018).

$$\%\;Alpha-amylase\;Inhibition=1-A\;\left(control\right)\;/\;A\;\left(sample\right)\times 100$$

### 2.8 Structural analysis

#### 2.8.1 High-performance liquid chromatography (HPLC)

Identification of novel bioactive compounds in the extract was accessed through HPLC analysis. Following standard protocols, 1 mL hydrochloric acid was mixed with 20 mL of ethanol, containing 1 g/L BHT and 0.5 g of dry material. Sonification was performed for 15 min after the reaction mixture was gently mixed. The mixture was refluxed in a thermostat at 90°C for 2 h. A total of 20 µL of the sample was inserted, and measurements were at 280 nm (Khezeli et al., 2016).

#### 2.8.2 Fourier transform infrared spectroscopy (FTIR)

Fourier transform infrared spectroscopy was performed to identify different functional groups in the *C. zeylanicum* bark. After reducing, extracts in chloroauric solution were centrifuged at 10,000 rpm for nearly 15 min. To remove any unwanted protein/enzymes, the pellet was washed with deionized water three times. After that, materials were left to dry and crushed completely in the pellet mill. FTIR analysis was carried out through an Agilent Cary 630 FTIR model (Alizadeh Behbahani et al., 2020).

### 2.9 Statistical analysis

Data were analyzed through ANOVA (one-way variance analysis) to calculate the average of two populations among nanosuspensions and extract. The measured data was recorded as average, percentage (%), and standard deviation (SD). A *p*-value less than 0.05 revealed the significance among nanosuspensions and extract.

## 3 Results

### 3.1 Antioxidant estimation

*In-vitro* antioxidant potential of *C. zeylanicum* nanosuspensions and extract are presented in Table 1. Nanosuspension and extract showed TPCs values of 39.51 ± 0.008 and 341.88 ± 0.31 mg GAE/100 g. While total flavonoid content (TFCs) in barks nanosuspension and extract were 239.26 ± 3.89 and 429.19 ± 0.07 mg CE/100 g, respectively. Extract and nanosuspension of *C. zeylanicum* showed maximum radical scavenging activity at 27.3 ± 1.35 and 10.6 ± 1.35, respectively.

**TABLE 1:** Comparison of different assays of *C. zeylanicum* nanosuspension and extract.

Treatments	Antioxidant profile			Antidiabetic profile (%)		Biofilm inhibition (%)		Cytotoxicity (%)
	TPC	TFC	DPPH	Glycation inhibition	α-amylase inhibition	EC	SA	
CNS	39.51 ± 0.008	239.26 ± 3.89	10.6 ± 1.35	42.14	6.3	66.09	26.89	19.89
CE	341.88 ± 0.31	429.19 ± 0.07	27.3 ± 1.35	53.76	39.3	67.11	-	54.78
Control	750.87 ± 6.63	244.44 ± 2.63	89.56 ± 0.0	56.91	82.53	59.39	42.01	96.45

* Results are represented as a percentage or as the mean and standard deviation of measurements taken in triplicate. CNS stands for Cinnamomum zeylanicum nanosuspension, CE stands for Cinnamomum zeylanicum extract, and *EC*: Escherichia *coli. SA*: *Staphylococcus aureus*. TPC: Total phenolic contents, TFC: Total flavonoid content, DPPH: 2,2-diphenyl l-picrylhydrazyl., ciprofloxacin (antimicrobial assay)., metformin (antiglycation assay)., and BHT (butylated hydroxytoluene).

### 3.2 Antiglycation activity

Antioxidant potential of *C. zeylanicum* nanosuspensions and extract are presented in Table 1. Nanosuspension and extract shows the antiglycation potential of 42.14% and 53.76%, respectively. One-way ANOVA shows a highly significant difference (*p* < 0.01) between the antiglycation analysis of cinnamon bark extract and nanosuspensions.

### 3.3 Alpha-amylase inhibition

The results of the alpha-amylase inhibition of *C. zeylanicum* barks extract and nanosuspensions were showed in Table 1. Cinnamon extract and nanosuspension showed alpha-amylase inhibition activity of 39.3% and 6.3%, respectively.

### 3.4 Biofilm inhibitory potential

**FIGURE 1:**
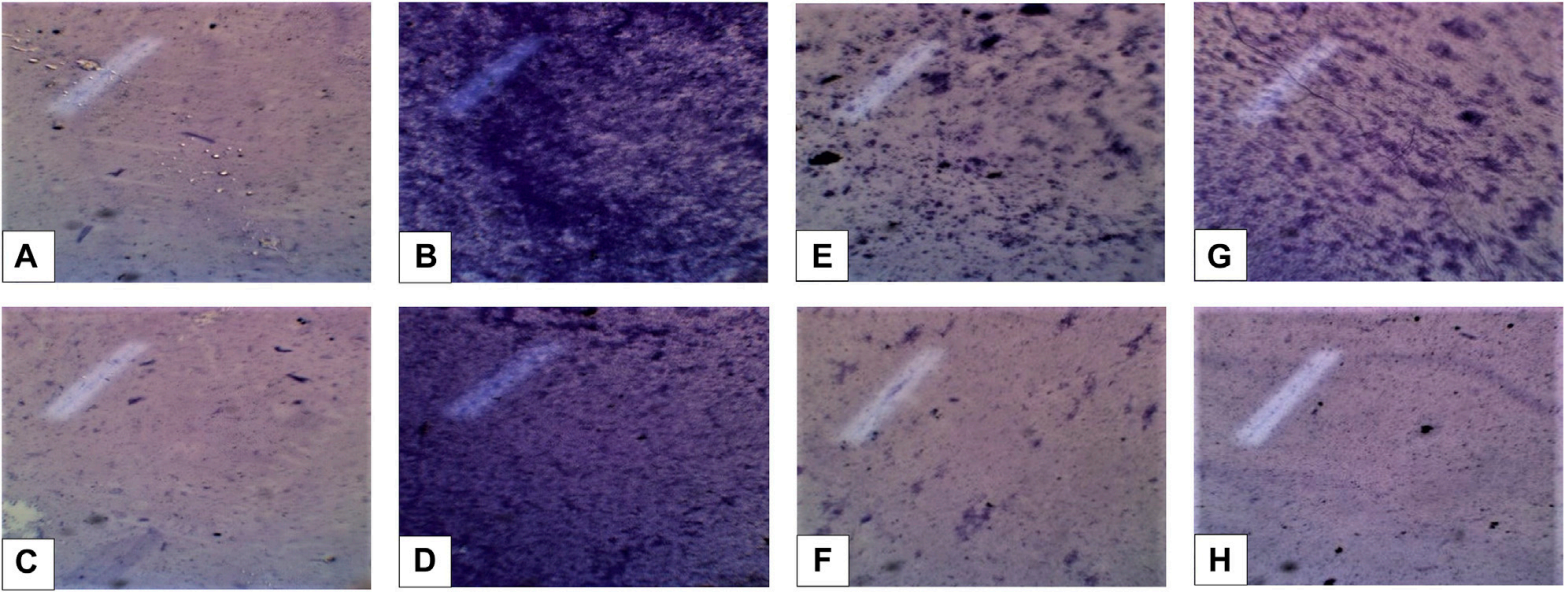
**(A,B)** (*Escherichia coli* positive and negative control) **(C,D)** *Staphylococcus aureus* positive and negative control. **(E,F)** Inhibition formation against *Escherichia coli* extract (min), nanosuspension (max). **(G,H)** Inhibition formation against *Staphylococcus aureus* extract (min), nanosuspension fraction (max).

Biofilm inhibitory potential of *C. zeylanicum* extract and nanosuspension was shown in Table 1 and Figure 1. *C. zeylanicum* extract and nanosuspension shows biofilm inhibition against *E. coli* (67% and 66.09%), respectively. While *C. zeylanicum* extract did not show any inhibitory activity against *S. aureus* and nanosuspension shows inhibitory activity of 26.89%. Cinnamon barks extract and nanosuspension strongly inhibits adhesion and biofilm formation. There is a highly significant difference (*p* < 0.01) between the inhibitory potential of cinnamon bark extract and nanosuspensions.

### 3.5 Cytotoxic activity

Cytotoxic potentials of *C. zeylanicum* extract and nanosuspensions are presented in Table 1. Results revealed that nanosuspension showed maximum hemolysis at 19.89%. However, *C. zeylanicum* bark extract resulted in 54.78% hemolysis. There is a statically highly significant (*p* < 0.01) difference between *C. zeylanicum* barks and nanosuspensions.

### 3.6 High-performance liquid chromatography (HPLC)

**FIGURE 2:**
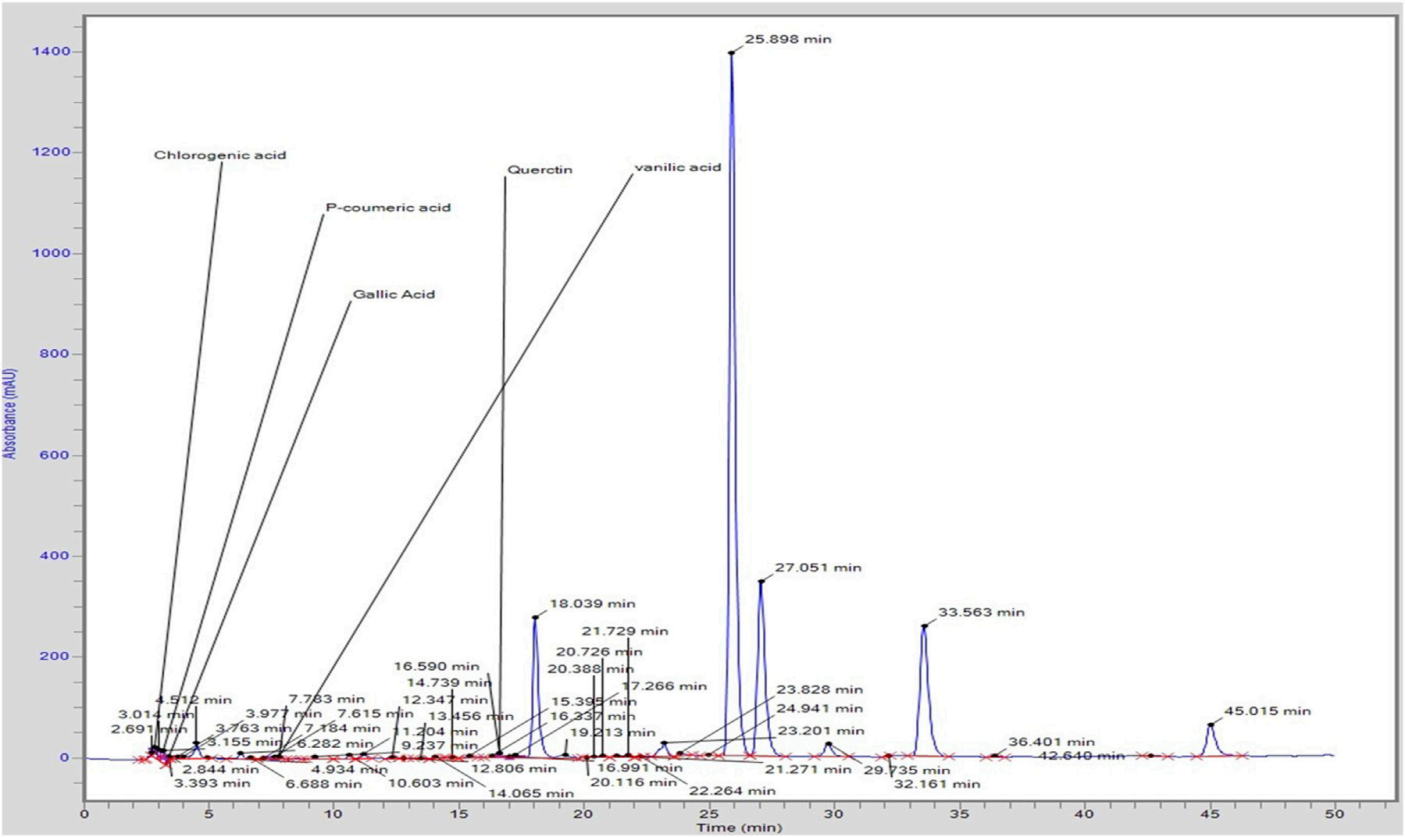
HPLC profile of *C. zeylanicum* bark extract.

Chromatogram generated from HPLC revealed the different peaks of compounds that exist in *C. zeylanicum* **(** Figure 2 **)**. HPLC analysis revealed that one flavonoid known as quercetin was detected. Whereas phenolic compounds were detected and identified as vanillic acid, gallic acid, p-coumaric acid and chlorogenic acid and. Table 2 shows the amount of the identified flavonoids and phenolic compounds. The flavonoid compound quercetin has (0.8 ppm), whereas; phenolic compounds gallic acid, chlorogenic acid, p-coumaric acid and vanillic acid have 0.34, 1.74, 28 and 0.5 ppm, respectively.

**TABLE 2:** Quantification of different flavonoids and phenolic compounds.

No. of compounds	Retention time	Height	Area	Amount (ppm)	Compound name	Nature of compound
1	2.844	20,295.0	154,093	1.74	Chlorogenic acid	Phenolic
2	3.155	23,792.9	233,808.2	28	p-Coumaric	Phenolic
3	3.393	8,690.9	39,211.4	0.34	Gallic acid	Phenolic
4	7.783	5,472.4	69,108.7	0.5	Vanillic acid	Phenolic
5	16.590	8,231.8	12,898.2	0.8	Quercetin	Flavonoid

### 3.7 Fourier-transform infrared (FTIR) spectroscopy

**FIGURE 3:**
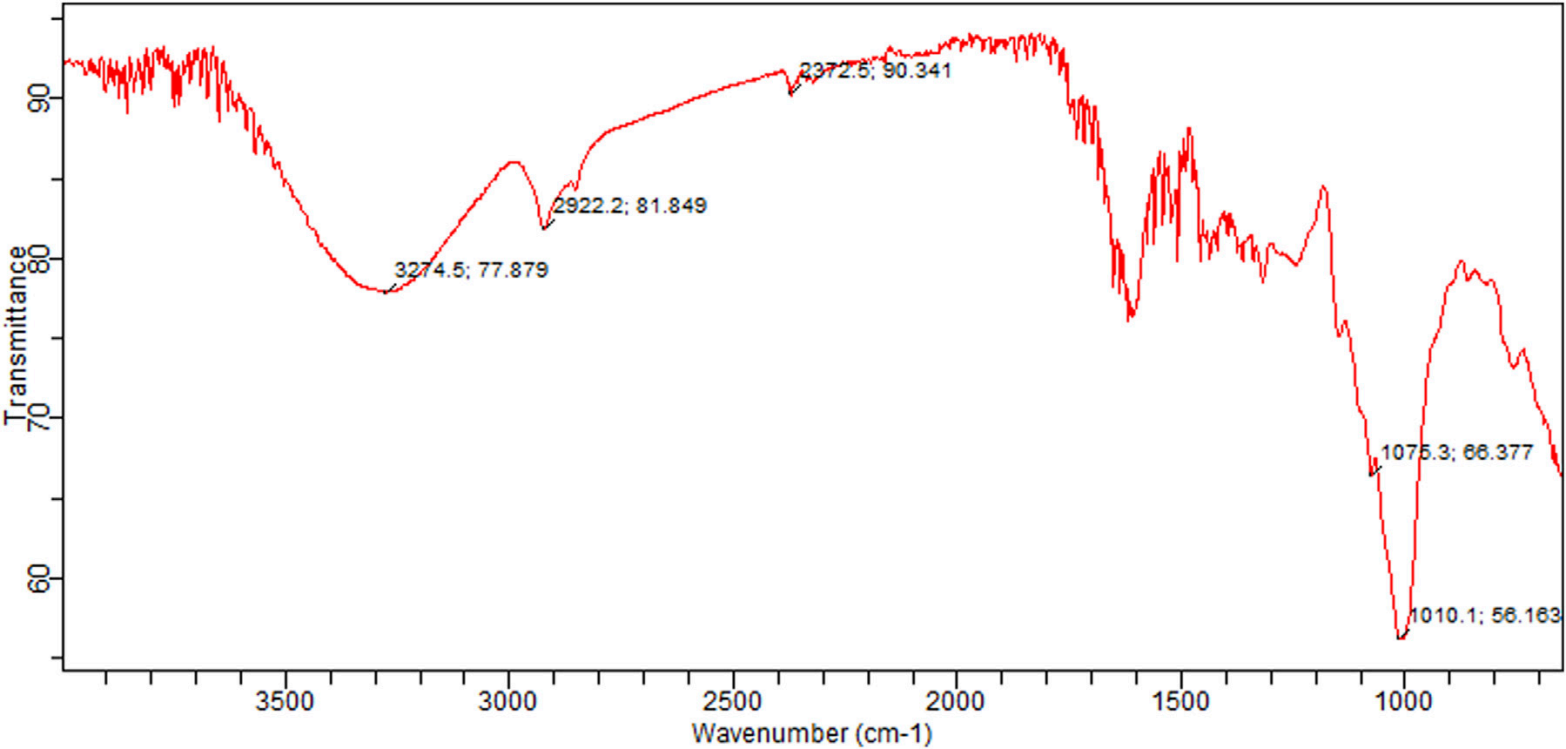
FTIR spectra of *C. zeylanicum* powder.

Graphical configuration of components found in *C. zeylanicum* and values depict the FTIR spectrum and absorption concentrations as various functional groups present in the cinnamon extract were shown in Table 3 and Figure 3. Alcohols were detected by a peak at 3,274 cm^−1^. Amine salts in the sample were indicated by a band at 2,922 cm^−1^. The existence of carbon dioxide is indicated by a band at 2,372 cm^−1^. Vinyl ether anhydride was detected by two intermediate bands at 1,075 cm^−1^ and 1,010 cm^−1^, respectively.

**TABLE 3:** FTIR spectrum chart representing the recognized functional groups in *C. zeylanicum.*

No. of compounds	Absorption bands	Recognized functional groups	Compounds
1	3,274	O-H stretches	Alcohol
2	2,922	N-H stretches	Amine Salt
3	2,372	O=C=O stretches	Carbon dioxide
4	1,075	C-O stretches	Vinyl ether
5	1,010	CO-O-CO stretches	anhydride

## 4 Discussion

Our findings are agreed with the previous studies (Husain et al., 2018; Singh et al., 2020; Madushika Wariyapperuma et al., 2021). Wickramasinghe et al. (2018) reported that methanolic nanosuspensions and extract of cinnamon bark contained a high amount of TPCs (27.64 ± 2.70 mg GAE/g). Other study by Wijewardhana et al. (2019) investigated that ethanolic extract and nanosuspensions of cinnamon bark showed TPCs of 18.94 mg GAE per 100 g of dry weight. Madushika et al. (2021) reported high TPCs in cinnamon extracts and nanosuspensions (20.87 ± 0.32 mg GAE g^−1^). In the present study, barks extracts showed higher TPCs than in the previous studies. Singh et al. (2020) reported that the nanosuspensions of *C. zeylanicum* showed the presence of TFCs (117.5 mg QE/g). Another study by Abeysekera et al. (2019) revealed that nanosuspensions and ethanolic extract of *C. zeylanicum* exhibited TFCs varied from 0.85 ± 0.01 to 4.68 ± 0.06 mg quercetin equivalents/g of the dry weight of the sample.

DPPH is a stable radical widely used to estimate free radical scavenging assay in many plant extracts. A recent study by Madushika Wariyapperuma et al. (2021) investigated the cinnamon extracts and silver nanoparticles showed high free radical scavenging activity at a concentration of 0.009 mg/mL. Another study reported by Singh et al. (2020) investigated that ethanolic extract and nanosuspensions of *C. zeylanicum* showed 87.33% ± 0.42% free radical scavenging activity at 1,000 μg/mL concentration. Our findings are consistent with the previous studies.

According to Zare et al. (2019), Cinnamon enhanced insulin sensitivity by increasing the insulin receptor kinase expression by suppressing insulin receptor dephosphorylation. A study reported by Wickramasinghe et al. (2018) investigated that 80% methanolic extract of *C. zeylanicum* showed 80% alpha-amylase inhibitory activity. Wariyapperuma et al. (2021) found that cinnamon extracts inhibited α-glucosidase (36 ± 8 μg mL^−1^) and α -amylase (57 ± 8 μg mL^−1^) activity. Hayward et al. (2019) demonstrated that Cinnamon’s showed anti-hyperglycemic properties and was more effective for diabetic patients. They revealed that cinnamon extract and nanosuspension showed alpha-amylase inhibitory activity at 82.53 ± 1.52 and 6.3 ± 5.13, respectively. Bark extracts had significantly higher anti-amylase activity when compared to nanosuspension and moderate when compared to the reference drug acarbose. Nanosuspensions in our study also exhibited antimicrobial potential and agreed with the previous studies.

A study by Anjum et al. (2019) investigated that ethanolic extract of cinnamon nanoparticles demonstrated significant antimicrobial activities. The inhibitory zone formation against *E. coli* and *S. aureus* were 4.23 0.5 mm and 3.21 0.09 mm, respectively. Abdulrasheed et al. (2019) reported that *E. coli* and *S. aureus* had the maximum while minimum susceptibility to cinnamon extract at 26.5 mm and 20 mm. Husain et al. (2018) reported that an ethanolic extract and nanosuspensions of Cinnamon had a maximum zone of biofilm formation (3.5 mm) against *S. aureus* at 10 mg/mL but no inhibition against *E. coli* at any intensity.

*In-vitro* cytotoxicity activity was performed to access the hemolytic potential of cinnamon extract. Husain et al. (2018) reported that cinnamon extract and nanosuspensions showed cytotoxic activity against MDA cells with an IC_50_ value of 25 g/mL. Another study by Najar et al. (2019) reported that *C. zeylanicum* EO was found to be effective against all the cell lines along IC_50_ at 20 ppm, while it was more effective on K562with IC_50_ value at 6 ppm and less effective on T47D (IC_50_ at 56.1 ppm). While Wanakhachornkrai et al. (2020) revealed that cinnamon nanosuspension had no cytotoxicity on human fibroblast cells at 100 g/mL concentrations, while 150 g/mL caused cytotoxicity.

Khalisyaseen and Mohammed (2021) reported the HPLC analysis of ethanolic extract-based nanosuspensions of *C. zeylanicum* bark and revealed the cinnamaldehyde: 74.67 ppm, eugenol: 6.998 ppm quercetin: 42.687 ppm, lignin: 5.860 ppm) and some phenolic components concentration (kaempferol: 0.0122 ppm, gallic acid: 0.030ppm). Similarly, this study also showed the *C. zeylanicum* ethanolic bark extract bioactive compounds, recognized and quantified by HPLC analysis, that contain quercetin (0.8 ppm), gallic acid (0.34 ppm), chlorogenic acid (1.74 ppm), p-coumaric acid (28 ppm) and vanillic acid (0.5ppm) in varying quantities (Iwata 2022).

Sanei et al. (2021) reported the nanoformulation of *C. zeylanicum* essential oils (CZEO). CZEO’s FTIR spectrum revealed a broad band at 3,468 cm^−1^ for hydroxyl groups, peaks at 3061 cm^−1^ for C-H, peaks at 2,923 cm^−1^ for CH stretching, bands at 2,812 and 2,740 cm^−1^ for C-H of aldehyde, band in 1728 cm^−1^ for C=O, and peak at 1,671 and 1,624 cm^−1^ for carbonyl C=O group correlated to an aldehyde stretch vibration. These strong peaks indicated the presence of aldehydes and cinnamaldehyde in Cinnamon. The peak at 2,924 cm^1^ in the spectra of CZ nanoparticles relates to C-H stretching and a band at 1710 cm^−1^ represents C=O, carbonyl stretch (Rodrigues et al., 2022). The current study confirmed the absorption projected by the FTIR and identified various functional groups in cinnamon nanosuspensions.

## 5 Conclusion

This research was designed to access the biochemical characterization and improved bioactivities of *C. zeylanicum* nanosuspensions through a nanotechnology approach. Structural and biochemical characterization was evaluated through FTIR and HPLC analyses. Results revealed that *C. zeylanicum* extract and nanosuspensions showed TPCs (341.88 and 39.51 mg GAE/100 g) and TFCs (429.19 and 239.26 mg CE/100 g) and DPPH inhibition potential (27.3% and 10.6%) respectively. Biofilm inhibition activity revealed that barks extract and nanosuspension showed excessive growth restraint against *E. coli* up to 67.11% and 66.09%, respectively. Alpha-amylase inhibition assay of extract and nanosuspension was 39.3% and 6.3%, while the antiglycation activity of nanosuspension and extract was 42.14% and 53.76%, respectively. Extract and nanosuspensions showed maximum hemolysis at 54.78 and 19.89, respectively. It was concluded that nanosuspensions possessed antidiabetic, antimicrobial, anticancer and antioxidant properties. The findings of this research may be the potential for using ethanolic bark extract nanosuspension in treating infectious diseases and could be the attention of future studies. These nanosuspensions based formulations may open the door to new research for the improved bioavailability of plant-based bioactive molecules.

## Data Availability

The original contributions presented in the study are included in the article/Supplementary Material, further inquiries can be directed to the corresponding authors.

## References

[B38] Abdulrasheed M. Ibrahim I. H. Luka A. Maryam A. A. Hafsat L. Ibrahim S. (2019). Antibacterial effect of cinnamon (*Cinnamomum zeylanicum*) bark extract on different bacterial isolates. J. Environ. Microbiol. Toxicol. 7 (1), 16–20. 10.54987/JEMAT.V7I1.466

[B1] Abeysekera W. P. K. M. Arachchige S. P. G. Abeysekera W. K. S. M. Ratnasooriya W. D. Medawatta H. M. U. I. (2019). Antioxidant and glycemic regulatory properties potential of different maturity stages of leaf of ceylon cinnamon (Cinnamomum zeylanicum blume) *in vitro* . Evidence-based Complementary Altern. Med. 2019, 1–10. 10.1155/2019/2693795 PMC6668558 31396287

[B2] Ali T. Hussain F. Naeem M. Khan A. Al-Harrasi A. (2022). Nanotechnology approach for exploring the enhanced bioactivities and biochemical characterization of freshly prepared nigella sativa L Nanosuspensions and their phytochemical profile. Front. Bioeng. Biotechnol. 10, 888177. 10.3389/fbioe.2022.888177 35656198 PMC9152536

[B3] Alizadeh Behbahani B. Falah F. Lavi Arab F. Vasiee M. Tabatabaee Yazdi F. (2020). Chemical composition and antioxidant, antimicrobial, and antiproliferative activities of Cinnamomum zeylanicum bark essential oil. Evidence-based Complementary Altern. Med. 2020, 1–8. 10.1155/2020/5190603 PMC7210559 32419807

[B37] Anjum S. Jacob G. Gupta B. (2019). Investigation of the herbal synthesis of silver nanoparticles using *Cinnamon zeylanicum* extract. Emergent Mater. 2 (1), 113–122. 10.1007/S42247-019-00023-X/FIGURES/9

[B4] Chahardehi A. M. Ibrahim D. Sulaiman S. F. (2009). Antioxidant activity and total phenolic content of some medicinal plants in Urticaceae family. J. Appl. Biol. Sci. 3, 25–29.

[B5] Gahtori R. Tripathi A. H. Kumari A. Negi N. Paliwal A. Tripathi P. (2023). Anticancer plant-derivatives: Deciphering their oncopreventive and therapeutic potential in molecular terms. Futur J. Pharm. Sci. 9, 14. 10.1186/s43094-023-00465-5

[B6] Grifoni L. Vanti G. Donato R. Sacco C. Bilia A. R. (2022). Promising nanocarriers to enhance solubility and bioavailability of cannabidiol for a plethora of therapeutic opportunities. Molecules 27, 6070. 10.3390/molecules27186070 36144803 PMC9502382

[B39] Hayward N. J. McDougall G. J. Farag S. Allwood J. W. Austin C. Campbell F. (2019). Cinnamon shows antidiabetic properties that are species-specific: effects on enzyme activity inhibition and starch digestion. Plant Foods Hum. Nutr. 74 (4), 544–552. 10.1007/S11130-019-00760-8/FIGURES/5 31372918 PMC6900266

[B7] Husain I. Ahmad R. Chandra A. Raza S. T. Shukla Y. Mahdi F. (2018). Phytochemical characterization and biological activity evaluation of ethanolic extract of Cinnamomum zeylanicum. J. Ethnopharmacol. 219, 110–116. 10.1016/J.JEP.2018.02.001 29408310

[B8] Hussain F. Akram A. Hafeez J. Shahid M. (2021). Biofunctional characterization of red, black and white ginseng (Panax ginseng Meyer) root extracts. Rev. Mex. Ing. Quim 20, 175–186. 10.24275/rmiq/bio1735

[B9] Hussain Z. Khan J. A. Arshad A. Asif P. Rashid H. Arshad M. I. (2019). Protective effects of Cinnamomum zeylanicum L (Darchini) in acetaminophen-induced oxidative stress, hepatotoxicity and nephrotoxicity in mouse model. Biomed. Pharmacother. 109, 2285–2292. 10.1016/j.biopha.2018.11.123 30551486

[B40] Iwata G. J. S. (2022). High performance liquid chromatography analysis of cinnamon from different origin. Column. 18 (21), 18–21. 10.2903/J.EFSA.2008.793

[B10] Jacob S. Nair A. B. Shah J. (2020). Emerging role of nanosuspensions in drug delivery systems. Biomater. Res. 24, 3–16. 10.1186/s40824-020-0184-8 31969986 PMC6964012

[B11] Jahan N. Aslam S. Rahman K. U. Fazal T. Anwar F. Saher R. (2016). Formulation and characterisation of nanosuspension of herbal extracts for enhanced antiradical potential. J. Exp. Nanosci. 11, 72–80. 10.1080/17458080.2015.1025303

[B12] Jain C. Khatana S. Vijayvergia R. (2019). Bioactivity of secondary metabolites of various plants: A review. Int. J. Pharm. Sci. Res. 10, 494. Article in. 10.13040/IJPSR.0975-8232.10(2).494-04

[B13] Johnson D. B. Nebhan C. A. Moslehi J. J. Balko J. M. (2022). Immune-checkpoint inhibitors: Long-term implications of toxicity. Nat. Rev. Clin. Oncol. 19, 254–267. 10.1038/s41571-022-00600-w 35082367 PMC8790946

[B14] Khalisyaseen O. Mohammed M. T. (2021). “Identification of some antioxidant active compounds,” in True cinnamon (cinnamomm zeylanicum) bark extract (Turkey; NVEO-Natural Volatiles & Essential Oils Journal| NVEO), 7565–7577.

[B15] Khezeli T. Daneshfar A. Sahraei R. (2016). A green ultrasonic-assisted liquid–liquid microextraction based on deep eutectic solvent for the HPLC-UV determination of ferulic, caffeic and cinnamic acid from olive, almond, sesame and cinnamon oil. Talanta 150, 577–585. 10.1016/J.TALANTA.2015.12.077 26838445

[B16] Kowalska J. Tyburski J. Matysiak K. Jakubowska M. Łukaszyk J. Krzymińska J. (2021). Cinnamon as a useful preventive substance for the care of human and plant health. Molecules 26, 5299. 10.3390/MOLECULES26175299 34500731 PMC8433798

[B17] Ma Y. Cong Z. Gao P. Wang Y. (2023). Nanosuspensions technology as a master key for nature products drug delivery and *in vivo* fate. Eur. J. Pharm. Sci. 185, 106425. 10.1016/j.ejps.2023.106425 36934992

[B18] Madushika Wariyapperuma W. A. N. Kannangara S. Wijayasinghe Y. S. Subramanium S. Jayawardena B. (2021). Fungal pretreatment to enhance the yield of phytochemicals and evaluation of α-amylase and α-glucosidase inhibition using Cinnamomum zeylanicum (L) quills pressurized water extracts. Lett. Appl. Microbiol. 72, 196–205. 10.1111/LAM.13410 33030748

[B19] Matsuda H. Wang T. Managi H. Yoshikawa M. (2003). Structural requirements of flavonoids for inhibition of protein glycation and radical scavenging activities. Bioorg Med. Chem. 11, 5317–5323. 10.1016/j.bmc.2003.09.045 14642575

[B20] Mishra S. B. Pandey H. Pandey A. C. (2013). Nanosuspension of Phyllanthus amarus extract for improving oral bioavailability and prevention of paracetamol induced hepatotoxicity in Sprague-Dawley rats. Adv. Nat. Sci. Nanosci. Nanotechnol. 4, 035007. 10.1088/2043-6262/4/3/035007

[B21] Najar B. Shortrede J. E. Pistelli L. Buhagiar J. (2019). Chemical composition and *in vitro* cytotoxic screening of sixteen commercial essential oils on five cancer cell lines. Wiley Online Libr. 17. 10.1002/cbdv.201900478 31713998

[B22] Oktay A. N. Karakucuk A. Ilbasmis-Tamer S. Celebi N. (2018). Dermal flurbiprofen nanosuspensions: Optimization with design of experiment approach and *in vitro* evaluation. Eur. J. Pharm. Sci. 122, 254–263. 10.1016/j.ejps.2018.07.009 29981401

[B23] Oshiomame Unuofin J. Aderonke Otunola G. Jide Afolayan A. (2018). *In vitro* a-amylase, a-glucosidase, lipase inhibitory and cytotoxic activities of tuber extracts of Kedrostis africana (L) Cogn. Heliyon 4, 810. 10.1016/j.heliyon.2018 PMC6169336 30294692

[B24] Powell W. A. Catranis C. M. Maynard C. A. (2000). Design of self-processing antimicrobial peptides for plant protection. Lett. Appl. Microbiol. 31, 163–168. 10.1046/j.1365-2672.2000.00782.x 10972721

[B25] Rahman M. M. Bibi S. Rahaman M. S. Rahman F. Islam F. Khan M. S. (2022). Natural therapeutics and nutraceuticals for lung diseases: Traditional significance, phytochemistry, and pharmacology. Biomed. Pharmacother. 150, 113041. 10.1016/j.biopha.2022.113041 35658211

[B26] Rodrigues E. Silva S. Zhang Y. Tan Y. Reymick O. Ouyang Q. (2022). γ-Cyclodextrin-Encapsulated cinnamaldehyde for citrus preservation and its potential mechanisms against Penicillium digitatum. J. Fungi 2022 8, 1199. 10.3390/JOF8111199 PMC9696930 36422020

[B27] Sahar P. Ali T. Naeem M. Hussain F. (2022). Nanotechnology approach for exploring the enhanced bioactivities, biochemical characterisation and phytochemistry of freshly prepared Mentha arvensis L nanosuspensions. Phytochem. Anal, 1–9. 10.1002/PCA.3189 36453173

[B28] Sanei-Dehkordi A. Moemenbellah-Fard M. D. Sereshti H. Shahriari-Namadi M. Zarenezhad E. Osanloo M. (2021). Chitosan nanoparticles containing Elettaria cardamomum and Cinnamomum zeylanicum essential oils; repellent and larvicidal effects against a malaria mosquito vector, and cytotoxic effects on a human skin normal cell line. Chem. Pap. 75 (12), 6545–6556. 10.1007/s11696-021-01829-y

[B29] Sharifi-Rad J. Dey A. Koirala N. Shaheen S. El Omari N. Salehi B. (2021). Cinnamomum species: Bridging phytochemistry knowledge, pharmacological properties and toxicological safety for health benefits. Front. Pharmacol. 12, 600139. 10.3389/fphar.2021.600139 34045956 PMC8144503

[B30] Singh R. Parasuraman S. Kathiresan S. (2020). Antioxidant and antidiabetic activities of methanolic extract of bark of Cinnamomum zeylanicum in diabetic rats. Free Radicals Antioxidants 10, 16–23. 10.5530/FRA.2020.1.4

[B31] Wanakhachornkrai O. Banglao W. Thongmee A. Sukplang P. (2020). Determination of antioxidant, anti-aging and cytotoxicity activity of the essential oils from Cinnamomum zeylanicum. J. Microbiol. Biotechnol. food Sci. 10, 436–440. 10.15414/JMBFS.2020.10.3.436-440

[B32] Wickramasinghe W. T. H. C. Peiris L. D. C. Padumadasa C. (2018). Chemical and biological studies of value-added cinnamon products in the Sri Lankan market. Panchkula, India: Society of Pharmaceutical Science and Research. Available at: https://ijpsr.com/bft-article/chemical-and-biological-studies-of-value-added-cinnamon-products-in-the-sri-lankan-market/ .

[B33] Wijewardhana U. S. Gunathilaka U. G. S. A. Navaratne S. B. (2019). Determination of changes occurrence in chemical properties of antioxidant incorporated palm olein during deep-fat frying. J. Pharmacogn. Phytochemistry 8 (5), 351–354.

[B34] Zafar F. Jahan N. Khalil-Ur-Rahman Asi M. Zafar W. U. I. (2020). Nanosuspension enhances dissolution rate and oral bioavailability of Terminalia arjuna bark extract *in vivo* and *in vitro* . Asian Pac J. Trop. Biomed. 10, 164–171. 10.4103/2221-1691.280293

[B35] Zafar F. Jahan N. Khalil-Ur-Rahman Bhatti H. N. (2019). Increased oral bioavailability of piperine from an optimized piper nigrum nanosuspension. Planta Med. 85, 249–257. 10.1055/a-0759-2208 30357764

[B36] Zare R. Nadjarzadeh A. Zarshenas M. M. Shams M. Heydari M. (2019). Efficacy of cinnamon in patients with type II diabetes mellitus: A randomized controlled clinical trial. Clin. Nutr. 38, 549–556. 10.1016/j.clnu.2018.03.003 29605574

